# Association of Decreased Handgrip Strength with Reduced Cortical Thickness in Japanese Female Patients with Type 2 Diabetes Mellitus

**DOI:** 10.1038/s41598-018-29061-x

**Published:** 2018-07-17

**Authors:** Miyuki Nakamura, Masaaki Inaba, Shinsuke Yamada, Etsuko Ozaki, Saori Maruo, Senji Okuno, Yasuo Imanishi, Nagato Kuriyama, Yoshiyuki Watanabe, Masanori Emoto, Koka Motoyama

**Affiliations:** 1 Department of Metabolism, Endocrinology and Molecular Medicine, Osaka City University Graduate School of Medicine, Osaka, Japan; 2 Department of Epidemiology for Community Health and Medicine, Kyoto Prefectural University of Medicine, Kyoto, Japan; 3 Department of Nephrology, Shirasagi Hosiptal, Osaka, Japan

## Abstract

LD-100, a quantitative ultrasonic device, allows us to measure cortical thickness (CoTh). Patients with type 2 diabetes mellitus (T2DM) show high prevalence of sarcopenia. This study aimed to clarify the association of handgrip strength (HGS) with cortical porosis, a major risk for fracture of DM. CoTh and trabecular bone mineral density (TrBMD) at the 5.5% distal radius were assessed in T2DM female patients (n = 122) and non-DM female controls (n = 704) by LD-100. T2DM patients aged older 40 years showed significantly lower HGS and CoTh, but not TrBMD, than non-DM counterparts. Although HGS was significantly and positively correlated with CoTh and TrBMD in T2DM patients, multivariate analysis revealed HGS as an independent factor positively associated with CoTh, but not TrBMD, in T2DM patients, suggesting the preferential association of HGS with cortical, but not trabecular, bone component in T2DM female patients. In conclusion, the present study demonstrated an early decline of HGS in T2DM female patients as compared with non-DM healthy controls after the age of 40 years, which is independently associated with thinner CoTh, but not TrBMD in T2DM patients, and thus suggested that reduced muscle strength associated with DM might be a major factor for cortical porosis development in DM patients.

## Introduction

Evidence has been accumulated to indicate that patients with type 2 diabetes mellitus (T2DM) have a significantly higher rate of fracture than non-DM individuals for their bone mineral density (BMD), suggesting development of impaired bone quality related to T2DM^1,2^. Diabetic bone disease is characterized by a low rate of bone turnover resulting from osteoblast dysfunction caused by sustained high glucose exposure and/or insulin/insulin-like growth factor deficits^3,4^, resulting in the accumulation of micro-damage inside bone tissue due to impaired bone healing, as shown in an animal model treated with high-dose bisphosphonate^5^. Alternatively, increased advanced glycation end-products (AGEs) content within collagen crosslinks of bone may impair bone quality^6,7^.

Recent studies that used high-resolution peripheral quantitative computed tomography (HR-pQCT) have demonstrated a relationship of increased cortical porosity with higher incidence of bone fracture in T2DM patients^8,9^. Elderly T2DM patients have remarkable decreases in lean mass and muscle strength as compared with non-DM elderly individuals^10,11^. We previously reported that DM hemodialysis patients showed significantly weaker handgrip strength (HGS) than non-DM counterparts^12^, suggesting decreased HGS as an independent factor to associate with higher all-cause mortality in DM hemodialysis patients^13^. Furthermore, previous studies found a significant association of HGS with cortical thickness^14^.

Those findings prompted us to investigate whether T2DM female patients, as compared with non-DM healthy females, might exhibit weaker HGS, lower cortical thickness and trabecular BMD, which are determined with LD-100 apparatus, which allow us to determine cortical bone components separately from trabecular bone components^15^, and thus investigate the possible effect of reduced muscle strength on the development of cortical porosis in T2DM patients.

## Subjects and Methods

### Subjects

We enrolled consecutive Japanese T2DM patients (n = 122) who visited the Outpatient Clinic of the Department of Metabolism, Osaka City University Hospital, while non-DM healthy female subjects (n = 704) who participated in the Japan Multi-Institutional Collaborative Cohort Study in Kyoto were enrolled as a control group. To avoid gender differences in HGS and BMD and bone parameters by LD-100, the subjects in this study were restricted to females. Diagnosis of DM was based on a history of diabetes or according to the criteria of the American Diabetes Association^16^. For glycemic control, the patients were being treated with dietary therapy alone (13%), or an oral agent (sulfonylurea 28%, dipeptidyl peptidase-4 inhibitor 38%, glinide drug 2%, metformin 35%, glucagon-like peptide-1 receptor agonist 9%) or insulin (39%). Data regarding HGS and bone parameters were simultaneously obtained with the LD-100, and used for our analyses. Those who had acute illness, infection, or malignancy, or taking medications such as steroids or anti-osteoporotic drugs that might affect bone metabolism, were excluded from the study. None had metabolic bone disease, or another major diseases that might affect nutritional status or influence bone metabolism.

Written informed consent was obtained from all patients prior to participation in the present study. This cross-sectional investigation was approved by the Ethics Review Committee of Osaka City University Graduate School of Medicine (approval #164) and the Institutional Ethics Committee of Kyoto Prefectural University of Medicine (approval #RBMR-E-289), and was conducted in accordance with the principals of the Declaration of Helsinki.

### Clinical and laboratory measurements

Venous blood samples were obtained from the T2DM patients in the morning after an overnight fast and from the non-DM healthy subjects at the baseline of the J-MICC Study. Fasting plasma glucose (FPG), glycated hemoglobin (HbA1c), serum levels of calcium (Ca), phosphate (Pi), and creatinine (Cr) were measured with an enzymatic method with an auto-analyzer (Hitachi 7450; Hitachi Co., Tokyo, Japan). HbA1c was also measured in the non-DM normal subjects to negate the possibility of DM, while other data were obtained from medical records and questionnaire results. To assess renal function, eGFR was calculated using an equation for females proposed by the Japanese Society of Nephrology, as follows: eGFR (ml/minute per minutes/1.73 m^2^) = 194 × serum creatinine^−1.094^ × age^−0.287^ × 0.739.

### Measurement of HGS

HGS on the non-dominant side was measured with a hand dynamometer by experienced research staff blinded to all clinical and biochemical data. The patients were instructed to apply as much handgrip pressure as possible with their non-dominant hand. Measurements were repeated 3 times, with the highest score recorded in kilograms, as previously reported^12,13^.

### Measurements of bone densitometry and BMD by newly developed quantitative ultrasound (QUS) device, LD-100, and dual-energy X-ray absorptiometry (DXA)

Ultrasonic measurements of the non-dominant side of the 5.5% distal radius were performed by use of a newly introduced QUS device, the LD-100 system, which allows determination of the trabecular bone density (TrBMD), and cortical thickness (CoTh)^17–19^. Results obtained with that device have been validated by a significant correlation with values obtained with Stratec pQCT^17^. As we previously reported^15^, the LD-100 system consists of a pair of ultrasonic transducers located coaxially in the forward direction and is equipped with a computer system. The transducers move simultaneously for scanning, with one transmitting ultrasound waves through the objective region and the other receiving those ultrasonic signals. TrBMD is estimated by quantifying the attenuation of the ultrasound waves transmitted through bone and transmission velocity of the fast wave propagated through trabecular bone structures, respectively^15,19^. CoTh, which is expressed in millimeters, was estimated by analyzing the reflected and transmitted ultrasonic signals, as previously described^18,20^, though it is not possible to apply echo measurements to cortical thickness. This new QUS system has received approval for use as medical equipment in Japan. BMD in the T2DM patients was measured at the ultra-distal radius and distal radius 1/3 using a DXA device (QDR-2000; Hologic Inc., Waltham, MA), as we previously described^21^. LD-100-determined cortical thickness and TrBMD were expressed in mm, and mg/cm^3^, respectively, and DXA-determined BMD in mg/cm^2^. To avoid inter-examiner difference, bone parameters were determined by one examiner using the LD-100. The intra-observer differences (% coefficient of variation) for TrBMD and CoTh were 4.3% and 2.8%, respectively. To avoid the effects of age and gender on such bone parameters, each parameter was alternatively expressed as a Z-score, which is the number of standard deviation from the mean value for age-matched normal subjects of the same gender.

### Statistical analysis

Data were analyzed using the StatView 5.0 J program (Abacus Concepts, Inc., Berkeley, CA) or JMP 10 software (SAS Institute Inc., Cary, NC, USA). Values are presented as the mean ± SD, unless otherwise indicated. Differences between mean values for the diabetic patients and non-diabetic subjects were analyzed using Student’s *t*-test or Mann-Whitney’s U test. Correlation coefficients were calculated by simple regression analysis. Comparisons of two regression slopes were performed as previously described^22^. Multivariate linear regression analysis was used to assess the relationships between independent variables and bone parameters. A forward selection procedure was employed to select the best subset of independent risk variables and careful checks for possible bias were performed. For all statistical tests, a p value < 0.05 was considered to indicate statistical significance.

## Results

### Clinical and biochemical profiles of female T2DM patients and non-DM healthy controls

The baseline characteristics of the T2DM patients (n = 122) and non-DM healthy controls (n = 704) are shown in Table 1. The mean age of the patients was 62.3 ± 13.2 years, significantly older than that of the non-DM group (53.2 ± 10.0 years). The duration of T2DM was 11.5 years (5.0–20.0 years). HGS in the non-dominant hand was 17.0 ± 5.7 kg in the T2DM patients, significantly lower than that in the non-DM subjects (25.1 ± 5.4 kg). To avoid the effect of age on LD-100 bone parameters, the findings are expressed as a Z-score. The Z-score of CoTh determined at the 5.5% distal radius by the LD-100 was −0.48 (−1.38–0.38) in the T2DM patients, which was significantly lower than the respective value of −0.07 (−0.47–0.74) in the non-DM controls, while the DM patients showed a significantly higher TrBMD Z-score [0.14 (−0.58–0.80)] than in the non-DM group [−0.23 (−0.82–0.35)].

**Table 1:** Clinical and biochemical profiles of female T2DM patients and non-DM normal subjects.

	DM patients (n = 122)	Non-DM control (n = 704)	P value
Age (years)	62.3 ± 13.2	53.2 ± 10.0	<0.001
DM duration (years)	11.5 (5.0–20.0)	—	—
BMI (kg/m^2^)	26.9 ± 6.0	21.5 ± 3.1	<0.001
Hand grip strength (kg)	17.0 ± 5.7	25.1 ± 5.4	<0.001
Serum Creatinine (mg/dL)	0.68 (0.55–0.83)	0.61 (0.55–0.67)	<0.001
Serum Albumin (g/dL)	4.0 ± 0.4	4.5 ± 0.2	<0.001
eGFR (mL/min/1.73 m^2^)	64.7 (53.4–86.2)	79.3 (69.5–89.0)	<0.001
FPG (mg/dL)	123 ± 33	—	—
HbA1c (%)	8.4 ± 1.6	5.3 ± 0.3	<0.001
Serum Calcium (mg/dL)	9.3 (9.0–9.6)	—	—
Serum Phospohorus (mg/dL)	3.9 (3.7–4.2)	—	—
LD-100, 5.5% distal radius			
Trabecular BMD (Z-score)	0.14 (−0.58–0.80)	−0.23 (−0.82-0.35)	<0.001
Cortical thickness (Z-score)	−0.48 (−1.38 – 0.38)	−0.07 (−0.47–0.74)	<0.001

Data are expressed the mean ± SD or median (inter quartile range: IQR).

Difference between DM patients and non-DM control was examined by Mann-Whitney U test.

### Comparison of HGS and bone parameters between T2DM patients and non-DM controls after age stratification

**Figure 1:**
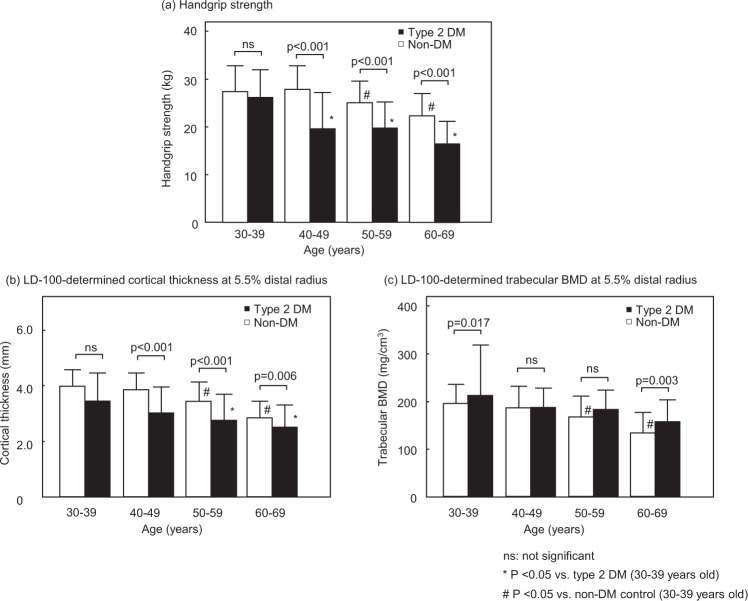
Age-stratified HGS (kg) (**a**) and LD-100-determined bone parameters CoTh (mm) (**b**) and TrBMD (mg/cm^3^) (**c**) at 5.5% distal radius in DM female patients (n = 122) and non-DM female controls (n = 704). HGS and cortical thickness were significantly lower in T2DM patients aged 40 years and older than in age-matched non-DM subjects, in contrast with no significant reduction in Tr.BMD in T2DM patients.

Figure 1 shows HGS and the LD-100-determined bone parameters CoTh and TrBMD at the 5.5% distal radius in the T2DM and non-DM groups after stratification by their age. HGS was significantly decreased in T2DM patients aged 40 years and older as compared with the same age group of non-DM controls (Fig. 1a). In addition, the T2DM patients aged 40 and older exhibited a reduction in CoTh that was significantly greater than that in the age-adjusted non-DM controls (Fig. 1b). Furthermore, an age-related decline became significant by their forties in T2DM patients, which was significantly earlier than the significant decline by fifties in non-DM controls (Fig. 1c). In contrast, no significant age-related decline in TrBMD was observed in T2DM patients from 30–69 years, although TrBMD significantly decreased in the non-DM controls aged 50–59 and 60–69 years as compared to those aged 30–39 years (Fig. 1c).

### Correlation of HGS with cortical bone components and trabecular bone components at the 5.5% distal radius in T2DM patients and non-DM subjects

**Figure 2:**
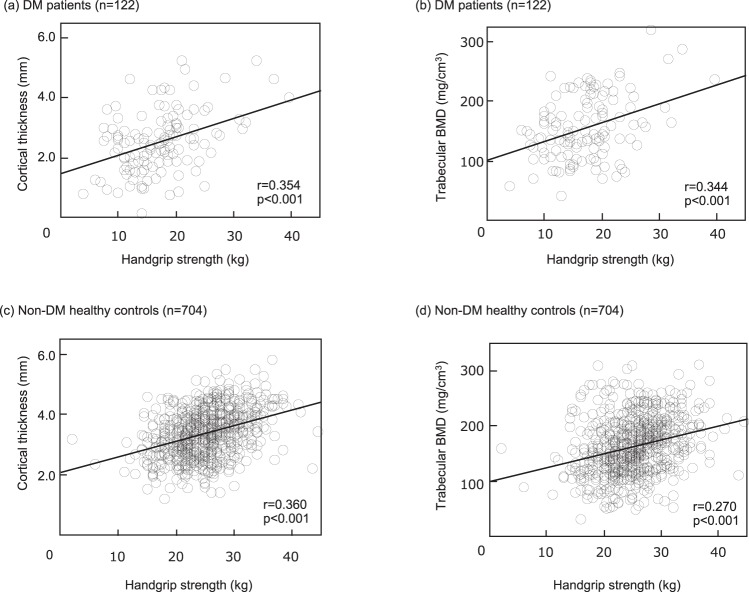
Correlation of HGS with CoTh (mm) and TrBMD (mg/cm^3^) at 5.5% distal radius in T2DM female patients (n = 122) and non-DM female controls (n = 704). HGS was significantly and positively correlated with CoTh (**a**: r = 0.354, p < 0.001) and TrBMD (**b**: r = 0.344, p < 0.001) in the T2DM female patients. HGS also showed a significant correlation with CoTh (**c**: r = 0.360, p < 0.001) and TrBMD (**d**: r = 0.270, p < 0.001).

Figure 2 shows the correlations of HGS with CoTh (mm) and TrBMD (mg/cm^3^) in the T2DM patients (n = 122) and non-DM controls (n = 704). HGS was significantly and positively correlated with not only CoTh (r = 0.354, p < 0.001) but also TrBMD (r = 0.344, p < 0.001) at the 5.5% distal radius in the T2DM patients. The non-DM subjects also showed a significant correlation of HGS with CoTh (r = 0.360, p < 0.001) as well as with TrBMD (r = 0.270, p < 0.001).

### Multivariate analysis to elucidate the independent factors associated with the Z-scores for CoTh and TrBMD at the 5.5% distal radius in T2DM patients

Next, we examined whether HGS was independently associated with the Z-scores for CoTh and TrBMD in the T2DM patients. Among the various clinical variables, CoTh correlated significantly in a negative manner with BMI (r = −0.169, p = 0.006), and in a positive manner with HGS (r = 0.354, p < 0.001), and serum albumin (r = 0.198, p = 0.029), while TrBMD was significantly correlated in a positive manner with BMI (r = 0.398, p < 0.001), and HGS (r = 0.344, p < 0.001), and tended to correlate positively with serum albumin (r = 0.176, p = 0.052).

As shown in Table 2, we also performed multivariate analysis which included BMI, HGS, eGFR, serum albumin, and HbA1c as independent variables. For independent variables selection, we selected the variables BMI and HGS, which showed a significant correlation with the CoTh Z-score, in addition to other variables known to affect bone mineral density in DM patients, i.e., eGFR, serum albumin, and HbA1c. Those results showed HGS to be an independent factor significantly and positively associated with the Z-score of CoTh, but not with that of TrBMD. Of interest, the same analysis elucidate BMI to be a significant factor positively associated with the Z-score of TrBMD, and negatively associated with the Z-score of CoTh.

**Table 2:** Multiple regression analysis to elucidate clinical variables associated with cortical thickness (Z-score) and trabecular BMD (Z score) in type 2 DM patients (n = 122).

	LD-100-determined CoTh (Z-score)		LD-100-determined TrBMD (Z-score)	
	β	p	β	p
BMI	−0.448	<0.001	0.217	0.025
Hand grip strength	0.319	0.001	0.125	0.223
eGFR	−0.191	0.051	−0.156	0.148
Albumin	0.163	0.075	0.061	0.543
HbA1c	0.097	0.286	−0.114	0.254
R^2^ (p value)	0.269 (<0.001)		0.111 (0.031)	

Shown are standard regression coefficient values (β values) and level of significance.

R^2^: coefficient of determination.

## Discussion

The present study demonstrated that HGS was significantly weaker in T2DM female patients aged 40 years and older than in non-DM female counterparts, and that LD-100-determined CoTh, but not TrBMD, was significantly thinner in the T2DM female patients aged 40 years and older than in non-DM female counterparts. Our finding revealed that HGS might be significantly and positively associated with LD-100-determined CoTh, but not TrBMD at 5.5% distal radius, indicating those to be clinically reliable indicators for cortical and trabecular bone component, respectively, in both T2DM female patients as well as non-DM female controls (Table 2). Also, it was suggested that a more prominent age-related decrease of HGS in DM female patients might contribute to thinner CoTh, which might reflect greater deterioration of cortical bone components in DM patients (Fig. 1).

Interestingly, using the same model for multiple regression analysis, BMI was shown to have an independent and negative association with CoTh, in contrast to its positive association with TrBMD (Table 2). We observed that T2DM female patients had a high prevalence of sarcopenic obesity along with high BMI for their lean mass than non-DM female controls. With the same muscle strength, as their BMI became higher in the DM patients, it seems that BMI may be negatively associated with CoTh. In contrast, it is likely that BMI is positively associated with TrBMD, as previously described^23^. This lack of influence of T2DM on trabecular bone components was noted in another study, which found that the trabecular microarchitecture shown by pQCT was not significantly different between postmenopausal T2DM females and an age- and race-matched control group^24^.

Another previous report found that T1DM and T2DM patients exhibited a higher fracture rate regardless of areal BMD findings determined by DXA^1^. Also, T2DM patients showed a 1.4-fold higher prevalence of osteoporotic fracture despite normal or elevated areal BMD^2,25^, suggesting that the increased fracture risk of this population cannot be explained by BMD, but rather by impaired bone quality or extra-skeletal factors such as hypoglycemia-induced falling, retinopathy, neuropathy, and renal osteopathy^26,27^. However, it is becoming increasingly recognized that DXA, a clinically useful tool for evaluating bone loss, has a serious limitation in regard to its lack of sensitivity to detect cortical deficits, such as increased cortical porosis and decreased cortical thickness^9^. On the other hand, the recently-developed high-resolution peripheral quantitative computed tomography (HR-pQCT) has emerged as an imaging modality that allows evaluations of three-dimensional cortical bone components separately from trabecular bone components *in vivo*^28^, and findings obtained with that technique have shown that patients with metabolic bone disease such as T2DM or CKD mainly lose cortical but not trabecular bone mass^8,9^.

It is known that cortical bone components play important roles in maintaining the axial load bearing capacity of long bones^29,30^, thus the amplitude of mechanical load on a long bone, which is mainly composed of cortical bone components, might determine its strength^31,32^. A previous study that utilized HR-pQCT demonstrated that impaired cortical bone components in particular increased cortical porosity and are responsible for fragility fractures in postmenopausal T2DM females^9^. Therefore, it is possible that deterioration of cortical bone components in patients with T2DM might lead to an increased incidence of fracture of long bones, such the femur and humerus, in affected patients^1^.

Diabetic osteopathy is characterized by osteoblast and osteocyte deficits, resulting in low bone turnover status. Bone is composed of dynamic tissues that respond to mechanical loads by adapting material properties through new bone formation^29^. However, it is possible that impaired bone formative activity due to a DM-induced osteoblast/osteocyte deficit might lower the adaptive response to mechanical loads, leading to bone fragility, particularly in cortical bone, which is involved in maintenance of long bone strength.

Although diabetic polyneuropathy has been reported to be an important factor in the development of diabetic sarcopenia^33,34^, the nerve damage associated with diabetic peripheral polyneuropathy, which is induced by metabolic effect, preferentially affects small fibers that transmit neural signal mainly in the autonomic and distal sensory pathway, leading to the progressive loss of sensation. Since the enrolled T2DM patients did not include those with advanced progression of DM complication, we confirmed that multiple regression analysis findings did not show an association of the reduced vibration sensation with HGS. Since vitamin D plays a major role in maintaining muscle strength simultaneously with bone mineral density^35,36^, it is possible that a vitamin D insufficiency or deficiency in T2DM patients contributes to reduced HGS and CoTh^37^.

Sarcopenia, which is frequently associated with DM^38^, and has been established as a main cause of bone fragility and fractures^39^. Insulin stimulates postprandial blood flow and glucose uptake into muscle cells, thus stimulating protein synthesis in muscle, suggesting that a relative or absolute insulin deficiency in DM patients is a factor related to development of sarcopenia^40^. Our findings showed an early age-related decrease in HGS in female T2DM patients. Furthermore, the significant relationship of age-related decrease in CoTh with age-related decreased in HGS in those patients as well as the non-DM healthy subjects indicate the significant contribution of early age-related decreases in those parameters in female T2DM patients. Sarcopenia has been recognized as an independent predictor of falling incidents and poor bone health^38,41^. Although obesity is generally considered to be associated with higher BMD, and thus acts as a protective factor against fracture, it is important to recognize that reduced HGS in individuals with coexisting obesity might act conversely to reduce CoTh in T2DM patients (Table 2).

This is the first study to demonstrate an association of early age-related decline in muscle strength with decreased cortical thickness in Japanese female T2DM patients, suggesting the importance of decreased muscle strength in development of cortical but not trabecular, bone deterioration in such patients. In addition, our findings raise the possibility that treatment to maintain muscle mass or strength might protect against the development of age-related loss of cortical bone strength in female T2DM patients as well as non-DM female healthy subjects. Although HR-pQCT has been established as the most sophisticated technique available to precisely measure multiple bone parameters, including trabecular and cortical bone components^28,42^, it was indicated that the widespread use of the newly introduced QUS device as a screening tool for cortical porosis without any exposure to X-ray may allow us to determine effective parameters for cortical bone separate from trabecular bone.

Our study has some limitations. First, cross-sectional design of the present study cannot depict causality. Furthermore, we focused only on reduced HGS in female T2DM and did not attempt to diagnose sarcopenia. Additionally, all of the subjects were Japanese, thus it is difficult to extend the results to other ethnicities. Finally, the present T2DM patients had received various drugs for DM, hypertension, and dyslipidemia, some of which might have effects on bone metabolism, thus potentially affecting the results.

In conclusion, the present study demonstrated early decline of HGS in T2DM female patients compared with non-DM healthy controls after the age of 40 years. In addition, that was shown to be independently associated with thinner CoTh, but not TrBMD in the T2DM patients, thus suggesting that reduced muscle strength associated with DM is a major factor associated with cortical porosis in DM patients.
